# MuSK cysteine-rich domain antibodies are pathogenic in a mouse model of autoimmune myasthenia gravis

**DOI:** 10.1172/JCI173308

**Published:** 2025-06-12

**Authors:** Marius Halliez, Steve Cottin, Axel You, Céline Buon, Antony Grondin, Léa S. Lippens, Mégane Lemaitre, Jérome Ezan, Charlotte Isch, Yann Rufin, Mireille Montcouquiol, Nathalie Sans, Bertrand Fontaine, Julien Messéant, Rozen Le Panse, Laure Strochlic

**Affiliations:** 1 Sorbonne Université, Institut National de la Santé et de la Recherche Médicale, Institut de Myologie, UMR-S 974 Centre de Recherche en Myologie, Paris, France.; 2 Sorbonne Université, Institut National de la Santé et de la Recherche Médicale, UMS28 Phénotypage du Petit Animal, Paris, France.; 3 Université Bordeaux, Institut National de la Santé et de la Recherche Médicale, Neurocentre Magendie, U1215, Bordeaux, France.; 4 Biochemistry and Biophysics Facility of the Bordeaux Neurocampus (BioProt), Bordeaux, France.

**Keywords:** Immunology, Muscle biology, Neuroscience, Autoimmune diseases, Neuromuscular disease, Skeletal muscle

## Abstract

The neuromuscular junction (NMJ), a synapse between the motor neuron terminal and a skeletal muscle fiber, is crucial throughout life in maintaining the reliable neurotransmission required for functional motricity. Disruption of this system leads to neuromuscular disorders, such as autoimmune myasthenia gravis (MG), the most common form of NMJ disease. MG is caused by autoantibodies directed mostly against the acetylcholine receptor (AChR) or the muscle-specific kinase MuSK. Several studies report immunoreactivity to the Frizzled-like cysteine-rich Wnt-binding domain of MuSK (CRD) in patients, although the pathogenicity of the antibodies involved remains unknown. We showed here that the immunoreactivity to MuSK CRD induced by the passive transfer of anti-MuSK^CRD^ antibodies in mice led to typical MG symptoms, characterized by a loss of body weight and a locomotor deficit. The functional and morphological integrity of the NMJ was compromised with a progressive decay of neurotransmission and disruption of the structure of presynaptic and postsynaptic compartments. We found that anti-MuSK^CRD^ antibodies completely abolished Agrin-mediated AChR clustering by decreasing the Lrp4-MuSK interaction. These results demonstrate the role of the MuSK CRD in MG pathogenesis and improve our understanding of the underlying pathophysiological mechanisms.

## Abstract 2

Immunoreactivity against an elusive domain of MuSK, as found in some Myasthenia Gravis patients, is pathogenic in an experimental mouse model

## Introduction

The electric signal inducing muscle contraction is transmitted via the neuromuscular junction (NMJ), a peripheral cholinergic synapse between a motor neuron’s highly specialized axon terminal and its skeletal muscle fiber target (1–3). Every step in NMJ formation and maintenance mainly relies on a complex of postsynaptic receptors composed of the muscle-specific kinase (MuSK) and its coreceptor, LDL receptor–related protein 4 (Lrp4) (4–7). This master complex for NMJ regulation acts as a scaffold for several secreted and intracellular partners required for efficient MuSK signaling. These partners include neural Agrin secreted by the motor neuron, which constitutes the principal molecular activator of MuSK, along with the Wnt morphogens that bind to MuSK via a Frizzled-like cysteine-rich domain (CRD) in its ectodomain, thereby initiating Wnt/MuSK signaling and participating in NMJ formation (8–13). In addition to the core Agrin/Lrp4/MuSK pathway, we previously demonstrated a role for Wnt-MuSK signaling at the NMJ, as MuSKΔCRD mutant mice present morphofunctional alterations of the entire neuromuscular system, including defects of acetylcholine receptor (AChR) clustering, a critical hallmark of postsynaptic differentiation of the synapse (14). In this context, disruption of the key molecular processes required for the precise alignment and differentiation of all components of the NMJ results in a loss of nerve/muscle connection, leading to highly debilitating neuromuscular disorders such as myasthenia gravis (MG).

MG is a severely weakening autoimmune disease caused by the production of autoantibodies against key NMJ proteins, reducing the stability of the NMJ and neurotransmission, and thus resulting in muscle fatigability. Most patients produce antibodies against the AChR but a small proportion (less than 10%) carry other antibodies, typically targeting MuSK (15). MuSK-MG is characterized by facial, cranial, and bulbar muscle weakness that can also affect the diaphragm, resulting in respiratory failure (16, 17). Symptomatic treatments for MuSK-MG include antiinflammatory corticosteroids and monoclonal immunotherapies such as rituximab (18, 19). In MuSK-MG, the NMJ is disrupted by immunoglobulins from the IgG4 subclass, excluding antigen cross-linking and complement cascade activation as pathogenic molecular events. Instead, anti-MuSK antibodies usually alter MuSK signaling by preferentially targeting the MuSK-Lrp4 interaction interface corresponding to the Ig-like domain 1 (Ig1) of MuSK. It is widely accepted that the MuSK Ig1 domain is the main immunogenic region, but several recent studies have described patients harboring not only anti–MuSK Ig1 domain antibodies, but also antibodies directed against the MuSK Ig2 domain and the MuSK CRD (20–22). The diversity of the epitopes targeted in these patients makes it difficult to assess the implications of antibodies against MuSK CRD (anti-MuSK^CRD^) in the development of the MG phenotype. The role of the MuSK CRD in NMJ pathogenesis remains largely unknown, and studies are therefore required to determine whether and how this particular domain of MuSK is involved in MG pathology.

We addressed this issue by generating mouse models of experimental autoimmune MG (EAMG) linked to the MuSK CRD through the passive transfer of rabbit polyclonal anti-MuSK^CRD^ antibodies or by active immunization with a mouse recombinant MuSK^CRD^ protein. We used an extensive array of behavioral, electrophysiological, and biochemical experiments to evaluate animal phenotypes and NMJ integrity and function. We found that anti-MuSK^CRD^ antibodies led to myasthenia-like symptoms accompanied by neurotransmission fatigability and severe morphological defects of the NMJ. Our results indicate that immunoreactivity to the MuSK CRD disrupted Agrin/Lrp4/MuSK signaling by decreasing the interaction between MuSK and Lrp4, thereby inhibiting Agrin-induced AChR clustering. This study, combining analysis of EAMG models in vivo and biochemical experiments in vitro, demonstrates the pathogenic role of the MuSK CRD in the context of MG and strengthens its implication in NMJ formation and stability.

## Results

### MuSK^CRD^ immunoreactivity leads to a myasthenic phenotype in mice.

**Figure 1:**
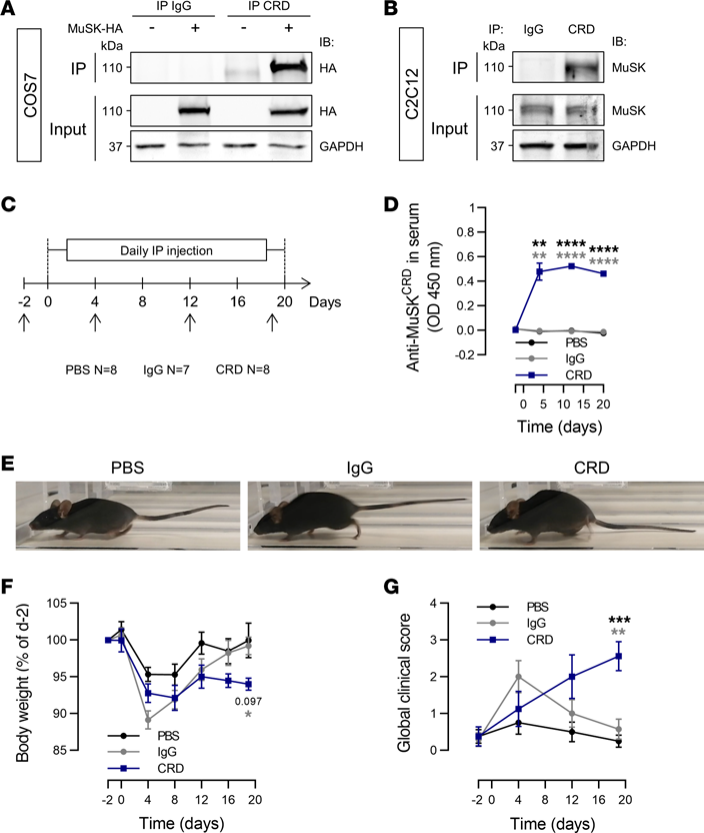
Anti-MuSK^CRD^ antibody injections induce myasthenia-like symptoms in mice. (**A** and **B**) Lysates of COS7 cells transfected with a MuSK-HA construct (**A**) and C2C12 myotubes (**B**) were subjected to IP with control IgG or anti-MuSK^CRD^ antibodies. Whole-lysate Western blots (Input) were performed to demonstrate the presence of the protein of interest. IB, immunoblot; GAPDH, loading control. (**C**) Schematic representation of the EAMG passive-transfer model protocol. PBS, control animals receiving injections of PBS; IgG, animals receiving injections of control IgG antibodies; CRD, animals receiving injections of anti-MuSK^CRD^ antibodies. Black arrows indicate the days on which clinical tests were performed. (**D**) ELISA for total anti-MuSK^CRD^ antibody dosing in serum from control (PBS and IgG) and immunized (CRD) animals (1:100,000) during the course of the protocol. (**E**) Representative images of PBS, IgG, and CRD animals running on a treadmill during the last clinical test session (day 19). (**F**) Body weight measurements for the PBS, IgG, and CRD groups during the course of the protocol. Weight was normalized relative to the value obtained during the preinjection clinical test (day –2). (**G**) Global clinical score (GCS) of PBS, IgG, and CRD animals during the protocol. GCS includes body weight, objective observation, hanging upside-down test, and grip test results. The data are shown as mean ± SEM. PBS, *n* = 8; IgG, *n* = 7; CRD, *n* = 8; *P < 0.05; **P < 0.01; ***P < 0.001; ****P < 0.0001. For **D**, **F**, and **G**, black statistical symbols are PBS versus CRD; gray statistical symbols are IgG versus CRD; 2-way ANOVA with Tukey’s post hoc test (**D**, **F**, and **G**).

Given the lack of biological samples from patients, we assessed the pathogenicity of antibodies targeting MuSK CRD by generating polyclonal MuSK^CRD^ IgG through immunization of rabbits with a peptide corresponding to the human MuSK CRD. We purified whole rabbit IgG from serum samples for use in subsequent in vivo experiments (hereafter referred to as anti-MuSK^CRD^ antibodies). We first assessed whether the anti-MuSK^CRD^ antibodies generated targeted MuSK in IP experiments on protein lysates from COS7 cells transfected with a MuSK-HA construct or C2C12-derived myotubes. No MuSK was detected in the fractions immunoprecipitated with control IgG, but a specific signal corresponding to the MuSK protein (110 kDa) was detected in both cell systems after IP with rabbit serum containing anti-MuSK^CRD^ (Figure 1, A and B). We then generated a mouse EAMG model by passive transfer with daily injections of anti-MuSK^CRD^ antibodies according to an immunization protocol adapted from Shen et al. (23) (Figure 1C). The immunized animals are referred to hereafter as CRD animals, and their counterparts receiving control injections of either PBS or nonspecific rabbit IgG are referred to as PBS or IgG animals, respectively. Anti-MuSK^CRD^ antibody titer was determined by ELISA on serum samples obtained on days –2, 4, 12, and 20. The amounts of anti-MuSK^CRD^ IgG in immunized animals remained stable from day 4 of the protocol onward (Figure 1D). Animals were monitored regularly throughout the experiment, and their motor functions were assessed. No behavioral or functional defects were observed in control PBS and IgG animals during the course of the protocol. During the last clinical testing session on day 19, the CRD animals rapidly became exhausted, displaying a spinal kyphosis and a trailing tail during a 5-minute treadmill run (Figure 1E). In all groups, the first injections induced a transient loss of body weight that persisted until day 8. Thereafter, PBS and IgG mice gained weight steadily throughout the rest of the protocol, whereas their CRD littermates initially regained weight and then started to lose weight again from day 12, with up to a 10% loss of body weight relative to the control animals on day 19 (Figure 1F). We measured overall symptom intensity by calculating a global clinical score, taking into account body weight loss, muscle strength in a grip test, objective observations of animal behavior, and the time spent hanging from an upside-down grid. PBS- and IgG-injected group scores were null during the experiment, reflecting an absence of myasthenic symptoms. By contrast, the global clinical score for the CRD animals increased progressively throughout the protocol and was significantly higher than that of the PBS animals at completion of the series of injections (PBS vs. IgG vs. CRD: 0.25 ± 0.2 vs. 0.57 ± 0.3 vs. 2.56 ± 0.4, Figure 1G). Thus, injections of rabbit sera containing IgG antibodies targeting MuSK CRD induce behavioral and motor deficits typical of myasthenia-like symptoms.

### Anti-MuSK^CRD^–injected mice display neurotransmission defects and neuromuscular fatigability.

**Figure 2:**
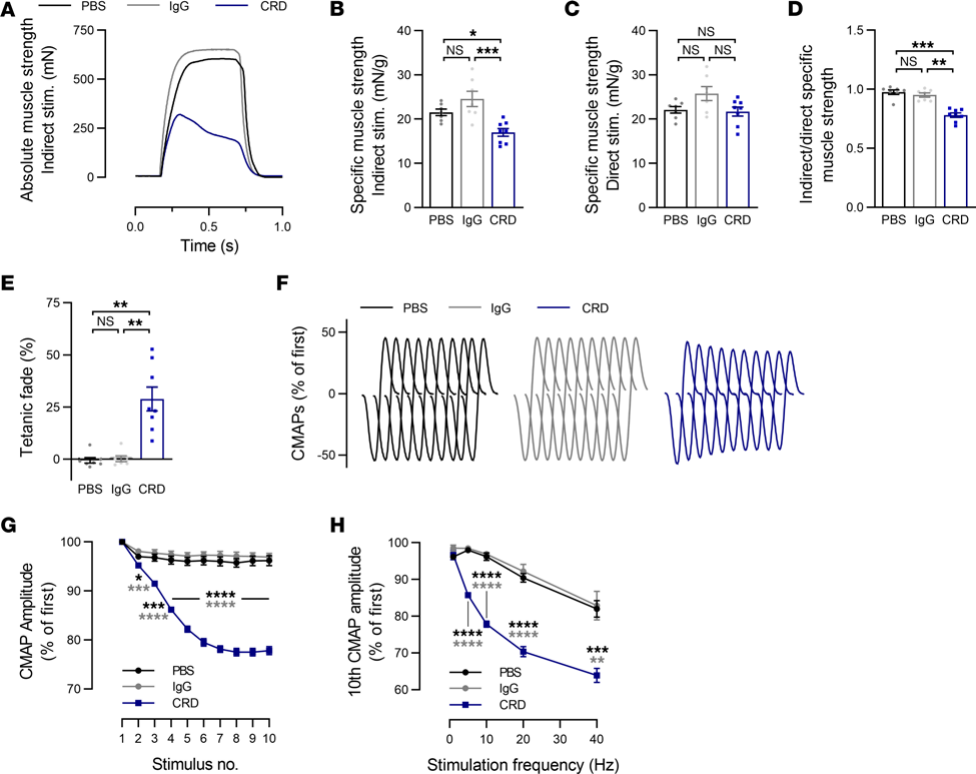
Changes to in situ muscle strength and reduced CMAP amplitude in mice injected with anti-MuSK^CRD^ antibodies. (**A**) Representative traces of maximal tibialis anterior (TA) muscle strength during tetanic stimulation of the sciatic nerve in controls (PBS and IgG) and an anti-MuSK^CRD^ antibody–immunized (CRD) animal. (**B** and **C**) Quantitative analysis of specific TA muscle strength corresponding to absolute maximal force relative to TA muscle mass after sciatic nerve (**B**) or TA muscle (**C**) stimulation. (**D**) Ratio of specific muscle strength between indirect and direct stimulations. (**E**) Quantitative analysis of the tetanic fade represented as the percentage of the maximal strength evoked during stimulation. (**F**) Representative traces of CMAPs from a PBS, IgG, and CRD animal. CMAPs were recorded in the TA muscle during a train of 10 sciatic nerve stimulations at 10 Hz. (**G**) Quantitative analysis of the CMAP amplitude after a 10 Hz stimulation, expressed as a percentage of the first CMAP amplitude. (**H**) Quantitative analysis of the amplitude of the 10th CMAP after trains of sciatic nerve stimulation at 1, 5, 10, 20, and 40 Hz. Data are expressed relative to the amplitude of the first CMAP of the train. The data are shown as mean ± SEM. PBS, *n* = 8; IgG, *n* = 7; CRD, *n* = 8; NS, not significant; *P < 0.05; **P < 0.01; ***P < 0.001; ****P < 0.0001. For **G** and **H**, black statistical symbols are PBS versus CRD; gray statistical symbols are IgG versus CRD; 1-way ANOVA with Tukey’s post hoc test (**B**–**E**) or 2-way ANOVA with Tukey’s post hoc test (**F** and **G**).

We investigated the effect of anti-MuSK^CRD^ antibodies on neurotransmission by performing in situ muscle strength recordings on the tibialis anterior after tetanic stimulation of the nerve (indirect) or muscle (direct) (Figure 2, A–C). Example traces of absolute strength developed during an indirect stimulation are shown in Figure 2A. The specific muscle strength, corresponding to the maximal force developed by the tibialis anterior divided by muscle mass, was significantly decreased during an indirect stimulation in CRD animals (PBS vs. IgG vs. CRD: 21.5 ± 0.7 vs. 24.6 ± 1.7 vs. 17.0 ± 0.9 mN/g, Figure 2B), whereas it was found equivalent in all groups during a direct stimulation (22.1 ± 0.8 vs. 25.7 ± 1.5 vs. 21.7 ± 1.0 mN/g, Figure 2C). Calculating the ratio of maximal force developed during an indirect stimulation over a direct stimulation revealed a significant decrease in CRD tibialis anterior strength compared with control groups (0.98 ± 0.02 vs. 0.95 ± 0.02 vs. 0.78 ± 0.02, Figure 2D), thus implying a neurotransmission defect instead of pure muscle weakness. We also quantified the tetanic fade, corresponding to the loss of strength during a tetanic stimulation, as displayed for a representative CRD mouse in Figure 2A. No tetanic fade was observed for the tibialis anterior from PBS and IgG animals, whereas a mean loss of 28.9% ± 5.7% of the maximal strength during stimulation was observed in CRD mice (Figure 2E). We investigated the integrity of neurotransmission further by recording compound muscle action potentials (CMAPs) on tibialis anterior muscle during trains of 10 stimulations of the sciatic nerve at different frequencies — 1, 5, 10, 20, and 40 Hz — and quantifying CMAP amplitudes. Peak-to-peak amplitude measurements showed that CMAP remained stable in PBS and IgG mice subjected to stimulation at 10 Hz, whereas these potentials gradually decreased in CRD animals stimulated in the same way (Figure 2, F and G). We determined the overall decrease observed at all frequencies tested by comparing the amplitude of the last CMAP of a train of stimuli with the amplitude of the first CMAP for the train concerned. CMAP amplitude in control groups was stable for stimulation at frequencies from 1 to 10 Hz and decreased by a total of 9.6% ± 1.2% and 7.8% ± 1.9% for a stimulation at 20 Hz in PBS and IgG animals, respectively. Similarly, for a 40 Hz stimulation, CMAP amplitude loss was quantified at 18.0% ± 2.3% for PBS animals and 17.1% ± 3.9% in the IgG group. In CRD mice, CMAP decrement was already significant from a 5 Hz stimulation. The total decrease was significantly greater by 18% than that in the control groups for stimulation at 10 Hz (96.2% ± 1.0% vs. 96.9% ± 0.8% vs. 77.9% ± 0.8%, Figure 2, G and H). Indeed, this decrease was exacerbated and greater than that in control animals for stimulations at higher frequencies, with a total decrease of 29.6% ± 1.4% at 20 Hz and 36.1% ± 1.9% at 40 Hz (Figure 2H). Taken together, these results demonstrate that anti-MuSK^CRD^ antibodies cause neurotransmission defects along with progressive neuromuscular weakness and fatigability in mice.

### Compromised NMJ morphological organization in anti-MuSK^CRD^–injected animals.

**Figure 3:**
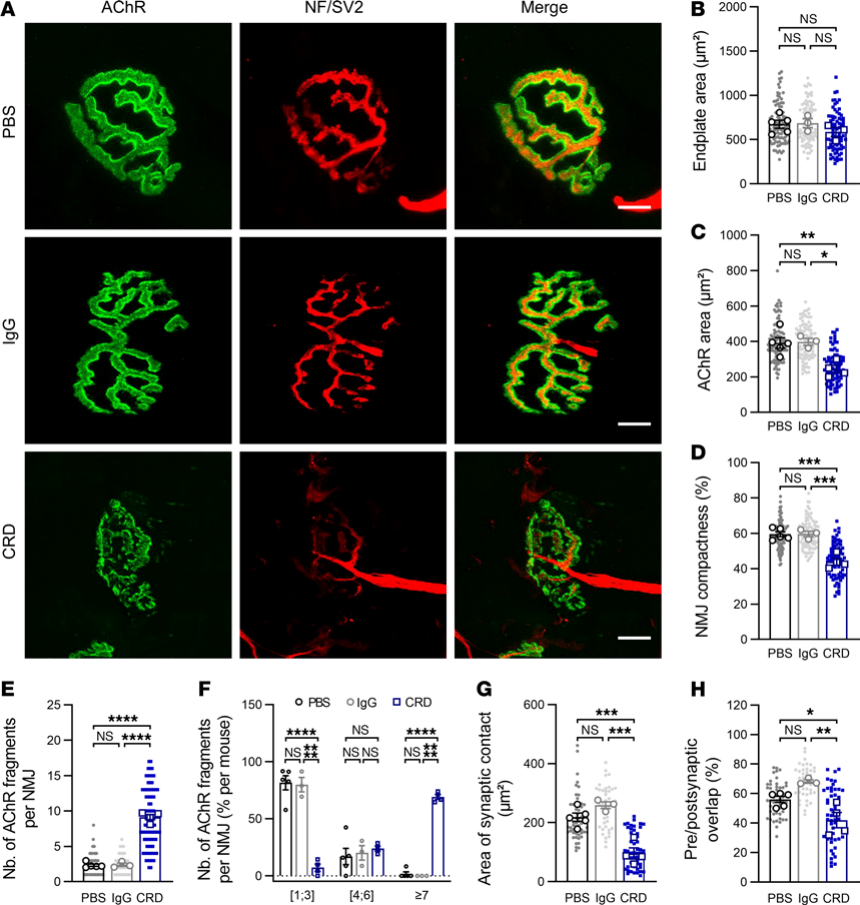
NMJ structural defects in the TA muscles of CRD mice. (**A**) Representative confocal images of isolated TA muscle fibers from control PBS, IgG, and CRD mice at the end of the passive immunization protocol. Muscles were stained with α-bungarotoxin for detection of the acetylcholine receptor (AChR, green) and antibodies directed against neurofilament and synaptic vesicle protein 2A (NF/SV2, red) for the labeling of nerve terminals. Scale bar: 10 μm in the merged images. (**B**–**H**) Quantitative analysis of the endplate area (**B**), AChR labeling area (**C**), NMJ compactness corresponding to the ratio of **C** to **B** (**D**), number of isolated AChR fragments per NMJ (**E**), distribution of NMJ by fragment number (**F**), area of contact between the nerve terminal and the area labeled for AChR (**G**), and the percentage of presynaptic and postsynaptic overlap corresponding to the ratio of **G** to **C** (**H**). The data are shown as mean ± SEM. PBS, *n* = 5; IgG, *n* = 3; CRD, *n* = 4. At least 20 NMJs were analyzed for each muscle. White-filled large symbols represent the mean of all NMJs for each biological replicate; ns, not significant; *P < 0.05; **P < 0.01; ***P < 0.001; ****P < 0.0001; 1-way ANOVA with Tukey’s post hoc test (**B**–**E**, **G**, and **H**), 2-way ANOVA with Tukey’s post hoc test (**F**).

We then explored whether the functional defects of the NMJ observed in CRD animals were correlated with structural alterations by analyzing NMJ morphological phenotypes for tibialis anterior muscles at the end of the EAMG protocol. Isolated muscle fibers were stained with fluorescent α-bungarotoxin to label AChR and with a mixture of antibodies against neurofilament and synaptic vesicle protein 2 to label nerve branches and terminals, respectively. As expected, the NMJ of PBS and IgG animals adopted a typical pretzel-like shape with a complex continuous network of AChR branches. Motor neuron labeling revealed a high degree of coverage of the postsynaptic apparatus by the nerve terminals. By contrast, in CRD mice, the AChR network displayed alterations with a loss of branch integrity and continuity along with the presence of numerous isolated AChR aggregates. Poor nerve-terminal coverage of the postsynaptic structures was also observed (Figure 3A). A quantitative analysis based on the workflow described by Jones et al. (24) showed that the endplate surface was similar in all groups (PBS vs. IgG vs. CRD: 673.0 ± 45.1 vs. 685.2 ± 51.4 vs. 572.3 ± 38.6 μm², Figure 3B), but AChR labeling within the endplates was significantly weaker in CRD animals (391.8 ± 30.4 vs. 397.5 ± 19.8 vs. 243.1 ± 21.5 μm², Figure 3C), resulting in a loss of NMJ compactness corresponding to the area of the AChR over the endplate area (59.5% ± 1.5% vs. 59.7% ± 1.6% vs. 44.0% ± 2.0%, Figure 3D). Indeed, the NMJ of CRD mice had 4 times the number of isolated AChR fragments found in control littermates (2.33 ± 0.2 vs. 2.41 ± 0.2 vs. 9.1 ± 0.4 fragments/NMJ, Figure 3E). Nearly none of the control NMJs analyzed consisted of more than 6 fragments, whereas highly fragmented synapses accounted for 69.1% ± 2.1% of all the NMJs analyzed in CRD animals (Figure 3F). There was a poor overlap between nerve terminals and AChR aggregates in the presence of anti-MuSK^CRD^ antibodies, as demonstrated by the significantly smaller area of synaptic contact (218.6 ± 13.7 vs. 259.1 ± 11.4 vs. 95.7 ± 19.4 μm², Figure 3G) and the overlap (i.e., the area of synaptic contact divided by the AChR area, 56.1% ± 2.2% vs. 68.2% ± 1.2% vs. 42.0% ± 5.2%, Figure 3H). Together, these results indicate that immunoreactivity to MuSK CRD leads to presynaptic and postsynaptic NMJ disassembly, consistent with the electrophysiological findings.

### Agrin-induced AChR clustering is inhibited by anti-MuSK^CRD^ antibodies.

**Figure 4:**
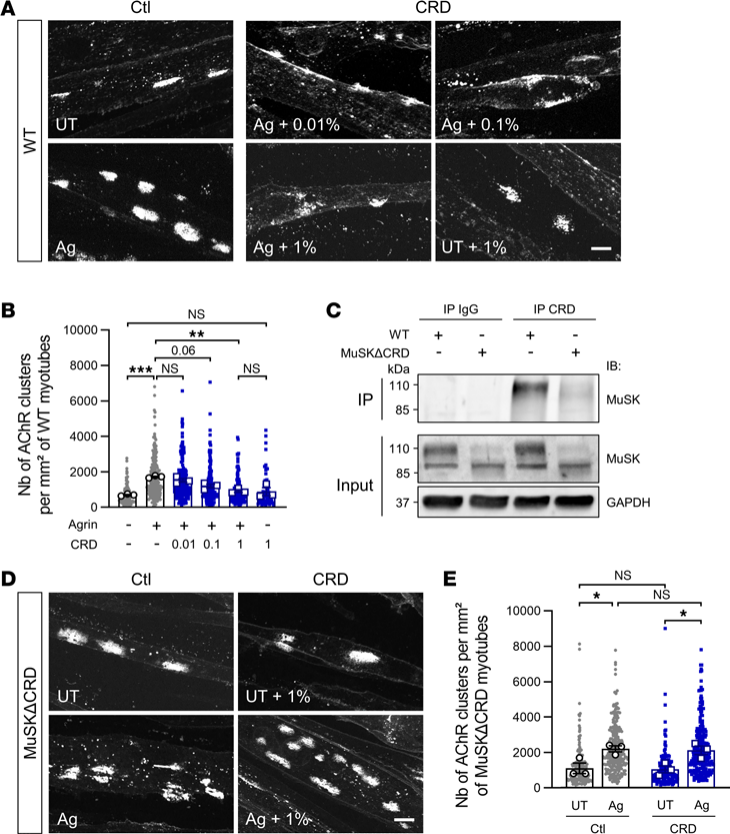
Anti-MuSK^CRD^ antibodies specifically inhibit Agrin-induced AChR clustering in vitro. (**A** and **D**) WT primary myotubes were treated with Agrin (Ag) together with increasing concentrations of MuSK^CRD^-immunized rabbit serum (CRD, %) or were left untreated (UT) for 16 hours (**A**). MuSKΔCRD primary myotubes were subjected to similar treatment with the optimal immunized rabbit serum concentration (1%, **D**). Cells were stained with α-bungarotoxin to label AChR clusters. Scale bar: 10 μm. (**B** and **E**) Quantitative analysis of the number of AChR clusters per mm² of myotube for **A** and **D**. (**C**) Lysates from WT and MuSKΔCRD primary skeletal muscle cultures were subjected to IP with control IgG or anti-MuSK^CRD^ antibodies. Whole-lysate Western blots (Input) were performed to demonstrate the presence of the proteins of interest. The specific MuSKΔCRD (85 kDa) band overlaps with a nonspecific signal observed in all lanes. IB, immunoblot; GAPDH, loading control. The data are shown as mean ± SEM. *n* = 3 independent experiments; at least 50 myotubes were analyzed for each treatment in each experiment. White-filled large symbols represent the mean of all the myotubes analyzed for each biological replicate; ns, not significant; *P < 0.05; **P < 0.01; ***P < 0.001; 1-way ANOVA with Tukey’s post hoc test (**B**), 2-way ANOVA with Tukey’s post hoc test (**E**).

AChR clustering at synaptic sites, a hallmark of postsynaptic differentiation, is principally dependent on Agrin/Lrp4/MuSK signaling; this pathway is crucial for NMJ formation, maturation, and maintenance (1). Agrin binds to Lrp4, enhancing the preexisting Lrp4-MuSK interaction and ultimately leading to MuSK activation and AChR aggregation. We investigated whether the NMJ defects observed in the passive EAMG model were due to changes in Agrin signaling. Primary myoblasts from WT mouse limb muscles were allowed to differentiate into myotubes and were then treated with Agrin, along with control or MuSK^CRD^-immunized rabbit serum. AChR aggregates were labeled with fluorescent α-bungarotoxin. Agrin treatment elicited a significant increase in the number of AChR clusters when cells were exposed to control rabbit serum (Ctl Agrin vs. Ctl untreated, 1,725.0 ± 38.5 vs. 700.8 ± 32.7 clusters per mm² of myotube, Figure 4A bottom left vs. top left and Figure 4B). However, AChR clustering was reset to basal levels when cells were treated with serum containing anti-MuSK^CRD^ antibody and Agrin (CRD Agrin + 1% vs. Ctl untreated, 918.2 ± 78.2 vs. 700.8 ± 32.7 clusters per mm² of myotube, Figure 4A bottom middle vs. top left and Figure 4B). Agrin-induced AChR clustering decreased progressively with increasing concentration of anti-MuSK^CRD^ antibodies, indicating a dose-dependent effect of serum. The spontaneous formation of AChR aggregates was not affected by immunized rabbit serum, suggesting that anti-MuSK^CRD^ antibodies specifically inhibit Agrin-induced clustering (CRD untreated + 1% vs. Ctl untreated, 884.1 ± 221.8 vs. 700.8 ± 32.7 clusters per mm² of myotube, Figure 4A bottom right vs. top left and Figure 4B). We further assessed the specificity of the anti-MuSK^CRD^ antibodies by performing IP experiments on extracts from WT or MuSKΔCRD primary muscle cultures with control IgG or immunized rabbit serum. As expected, a signal corresponding to MuSK was observed when WT lysates were subjected to IP with anti-MuSK^CRD^ antibodies. By contrast, these antibodies were unable to bind to MuSK from which the CRD had been deleted (Figure 4C). For confirmation of the specificity of the inhibition of the Agrin-induced AChR clustering mechanism by CRD immunoreactivity, we assessed the aggregation capacity of Agrin on MuSKΔCRD primary myotubes treated with immunized rabbit serum. In contrast to the results obtained for WT myotubes (Figure 4, A and B), AChR aggregation was similar in cells treated with Agrin and anti-MuSK^CRD^ antibodies and in cells treated with Agrin and control serum (CRD Agrin + 1% vs. Ctl Agrin, 2,124.0 ± 243.4 vs. 2,207.0 ± 170.4 clusters per mm^2^ of myotube, Figure 4D bottom right vs. bottom left and Figure 4E). This set of experiments indicates that antibodies against the MuSK CRD specifically and severely alter Agrin/Lrp4/MuSK signaling in vitro.

### Anti-MuSK^CRD^ antibodies decrease MuSK activation by inhibiting Lrp4-MuSK interaction.

**Figure 5:**
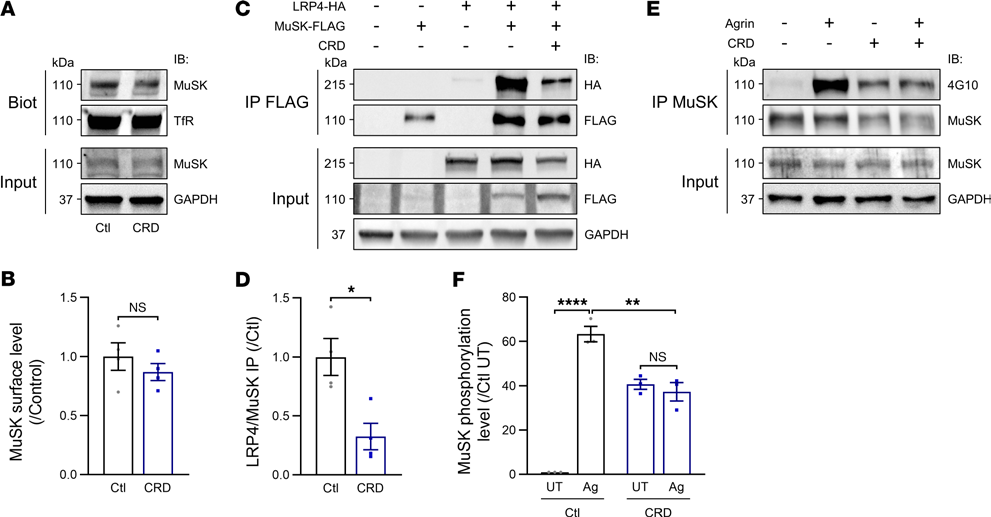
Molecular mechanisms of anti-MuSK^CRD^ antibodies in vitro. (**A**) Western blot of surface-biotinylated proteins (Biot) in C2C12 myotubes with (CRD) and without (Ctl) treatment with anti-MuSK^CRD^ antibodies for 16 hours. Whole-lysate Western blots (Input) were performed to demonstrate the presence of the proteins of interest. Ctl, control; IB, immunoblot; TfR, transferrin receptor; GAPDH, loading control. (**B**) Quantitative analysis of **A** showing the ratio of biotinylated MuSK to TfR normalized relative to Ctl conditions. (**C**) MuSK-FLAG and Lrp4-HA co-IP assay with FLAG Fab-Trap agarose in transfected COS7 cells. Cells were left untreated (Ctl) or were subjected to pretreatment with anti-MuSK^CRD^ antibodies (CRD) for 16 hours. (**D**) Quantitative analysis of **C**, corresponding to the ratio of IP Lrp4-HA to MuSK-FLAG normalized against the Ctl condition (lane 4 in **C**). (**E**) MuSK IP in C2C12 myotubes left untreated or treated with Agrin after 3 hours of pretreatment with anti-MuSK^CRD^ antibodies (CRD). The 4G10 anti-phosphotyrosine antibody was used to assess MuSK phosphorylation. (**F**) Quantitative analysis of **E** corresponding to the 4G10 signal divided by the IP MuSK signal normalized against Ctl conditions (Ctl untreated [UT]). The data are shown as mean ± SEM. *n* = 4 (**B** and **D**) and *n* = 3 (**F**) independent experiments; ns, not significant; *P < 0.05; **P < 0.01; ****P < 0.0001; Mann-Whitney *U* test (**B** and **D**) or 2-way ANOVA with Tukey’s post hoc test (**F**).

We then investigated the mechanisms underlying the alterations to Agrin signaling after treatment with anti-MuSK^CRD^ antibodies. Control experiments based on cell-surface protein biotinylation assays showed that MuSK expression on the cell membrane was not altered by anti-MuSK^CRD^ treatment (Figure 5, A and B). We then assessed the Lrp4-MuSK interaction in a heterologous system. COS7 cells were transfected with plasmids either encoding for a FLAG-tagged MuSK protein or HA-tagged Lrp4 protein along with cotransfected conditions. Interestingly, the presence of anti-MuSK^CRD^ antibodies decreased the interaction between the recombinant MuSK-FLAG and Lrp4-HA proteins significantly, by 68% relative to the control condition (0.32 ± 0.1 vs. 1.00 ± 0.2, Figure 5, C and D).

MuSK activation is known to be correlated with its level of tyrosine phosphorylation. We therefore assessed MuSK tyrosine phosphorylation using the 4G10 anti-phosphotyrosine antibody on IP MuSK from C2C12 myotube extracts. Agrin treatment alone resulted in high levels of MuSK tyrosine phosphorylation, whereas concomitant exposure with anti-MuSK^CRD^ antibody treatment resulted in significantly decreased levels of phosphorylation (lane 4 vs. lane 2, 41% lower; 37.4% ± 4.2% vs. 63.4% ± 3.5%, Figure 5, E and F). Overall, our data suggest that anti-MuSK^CRD^ antibodies disrupt Agrin/Lrp4/MuSK signaling by blocking the physical interaction between Lrp4 and MuSK, thereby impairing MuSK activation.

### MuSK^CRD^ active immunization does not recapitulate a neuromuscular passive transfer phenotype.

**Figure 6:**
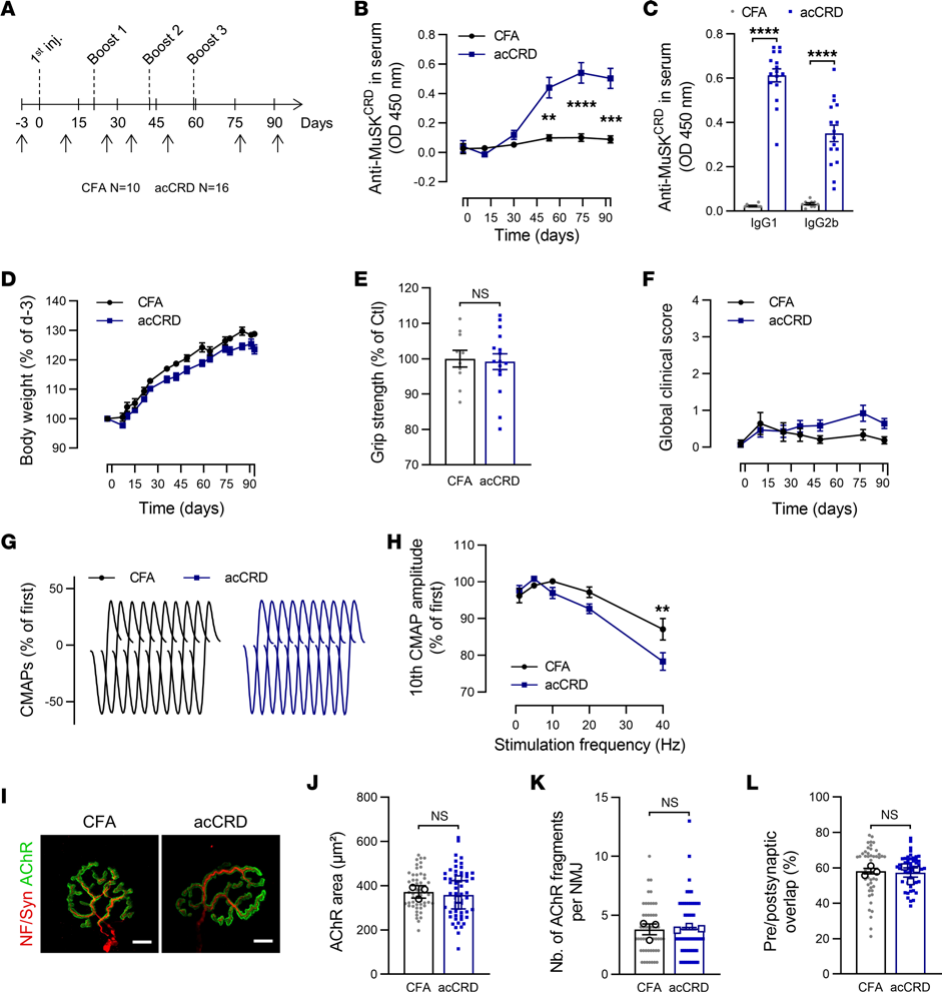
Animals immunized with MuSK^CRD^ peptide do not display myasthenia-like characteristics. (**A**) EAMG active transfer protocol. Black arrows indicate the days on which clinical tests were performed. (**B**) ELISA for total anti-MuSK^CRD^ antibody dosing in control (CFA, *n* = 10) and immunized (acCRD, *n* = 16) animal serum (1:1,000) during the protocol. (**C**) ELISA for anti-MuSK^CRD^ Ig1 and Ig2b antibody dosing in mouse serum (1:500) at the end of the immunization protocol. (**D**) Body weight normalized to the value obtained during the preimmunization clinical tests (day –3). (**E**) Forelimb muscle strength in the grip test in CFA and acCRD animals on day 91. Data are normalized relative to the CFA group. (**F**) Global clinical score for control and immunized animals. (**G**) Representative traces of CMAPs from TA muscles of a CFA and an acCRD animal after a train of 10 stimulations at 10 Hz. (**H**) Quantitative analysis and representation of CMAPs as described in Figure 2G. (**I**) Representative confocal images of isolated TA muscle fibers from CFA or immunized acCRD mice. Muscles were stained as described in Figure 3A, except that an anti-synaptophysin (Syn) antibody was used to stain nerve terminals. Scale bar: 10 μm. (**J**–**L**) Quantitative analysis of the area labeled for AChR (**J**), the number of AChR fragments per NMJ (**K**), and the percentage of presynaptic and postsynaptic overlap (**L**). The data are shown as mean ± SEM. *n* = 10 CFA and *n* = 16 acCRD for **A**–**H**. For **J**–**L,** *n* = 3 for each group, with at least 20 NMJs analyzed for each muscle. White-filled large symbols represent the mean of all the NMJs for each biological replicate (**J**–**L**); ns, not significant; **P < 0.01; ***P < 0.001; ****P < 0.0001; 2-way ANOVA with Tukey’s post hoc test (**B**, **D**, **F**, and **H**), Mann-Whitney *U* test (**C**, **E**, **K**, and **L**), and 2-tailed Student’s *t* test (**J**).

We generated a second EAMG model based on the active immunization of mice with the recombinant human MuSK CRD peptide to consolidate our results implicating anti-MuSK^CRD^ antibodies in MG pathogenesis. Mice were initially immunized with an injection of MuSK CRD peptide emulsified in CFA. They then received 3 booster injections on days 21, 42, and 59 with the MuSK CRD peptide emulsified in incomplete Freund adjuvant (these animals are referred to hereafter as acCRD animals). Control mice received initial and booster injections with emulsified PBS (these animals are referred to hereafter as CFA animals, Figure 6A). As expected, acCRD mice progressively developed anti-MuSK^CRD^ antibodies during the course of the protocol (Figure 6B). At the end of the immunization protocol, most of the antibodies against MuSK^CRD^ belonged to the Ig1 subclass, but Ig2b antibodies also accounted for a significant proportion of serum Ig (Figure 6C). Surprisingly, the immunization protocol did not induce any decrease in body weight or grip strength, and the global clinical score remained low, contrasting with the results obtained with the passive model (Figure 1 and Figure 6, D–F). Strength (data not shown) and CMAP electrophysiological recordings did not appear to differ from those for the control group (Figure 6G). The progressive decrease in CMAP amplitude was not significantly greater than that in PBS animals, except for high-frequency stimulations at 40 Hz (10th CMAP amplitude in percentage of the first, 78.3% ± 2.4% vs. 87.1% ± 2.9%, Figure 6H). The immunolabeling of NMJ on isolated tibialis anterior muscle fibers from both groups indicated that synapses displayed the expected structural complexity and continuousness (Figure 6I). Quantitative analyses further confirmed that the acCRD animals and their CFA littermates did not differ in terms of AChR labeling area, the number of AChR aggregates isolated, or coverage for presynaptic and postsynaptic signals (Figure 6, J–L). This active transfer immunization protocol based on injection of the MuSK^CRD^ peptide does not, therefore, elicit a behavioral, functional, or morphological myasthenic phenotype.

## Discussion

We showed here that antibodies targeting the MuSK CRD underlie the onset of a myasthenic phenotype accompanied by morphological and functional defects of the NMJ. Given the limited availability of biological material from the small number of patients carrying anti-MuSK^CRD^ antibodies together with other anti-MuSK antibodies, we investigated the pathogenic effect of MuSK^CRD^ antibodies by generating mouse EAMG models by the passive transfer of IgG antibodies purified from MuSK^CRD^-immunized rabbits or active immunization with the MuSK CRD peptide. Mice receiving injections of MuSK^CRD^ antibodies clearly reproduced several of the hallmarks of MG, consistent with the findings for other anti-MuSK IgG passive transfer models reported in previous studies (25). However, mice immunized with the recombinant MuSK^CRD^ peptide did not display the expected symptoms or NMJ dysfunction. According to the results from several previously described active immunization models for MuSK, MG initiation is variable and does not always occur (26, 27). In our active transfer model, the animals developed antibodies against the injected MuSK^CRD^ peptide but in quantities too small to trigger disease onset. The low levels of antibodies induced probably reflect the small amount of peptide injected or an inappropriate conformational structure, decreasing the immunogenic impact of the protein. However, genetic background has a well-documented role in the triggering of disease symptoms, and we cannot rule out a role for this factor here. An effect of genetic background is consistent with the highly variable nature of MuSK MG symptoms after active immunization, according to the mouse strain used. Our data indicate that the injection of the MuSK CRD peptide resulted in a greater decrease in CMAP for stimulations at a frequency of 40 Hz, suggesting that a stronger immune response would probably result in a phenotype similar to that observed in the passive EAMG model. Further studies are therefore required to establish a reliable model of MG with anti-MuSK^CRD^ antibodies by active immunization.

Our results indicate that the serum of MuSK^CRD^ peptide-immunized rabbits inhibited the interaction of Lrp4 with MuSK. This interaction is crucial for the essential Agrin/Lrp4/MuSK signaling pathway. This finding is consistent with the widely accepted principal pathological mechanism of antibodies against the MuSK Ig1 domain in human and animal models (28). However, it remains unclear whether the loss of Lrp4-MuSK binding in our model is due to steric hindrance or changes in the conformation of MuSK after antibody binding. We found that anti-MuSK^CRD^ treatment in vitro induced a partial loss of Agrin-induced MuSK phosphorylation and a total loss of Agrin-induced AChR clustering, with the cultured muscle cells harboring basal levels of AChR aggregates that could potentially account for the NMJ disease symptoms observed in vivo. Interestingly, exposure to anti-MuSK^CRD^ antibodies significantly increased MuSK phosphotyrosine levels relative to the control in the absence of Agrin. MuSK phosphorylation is considered to be proportional to the activation of MuSK and its ability to induce AChR clustering (29). The total loss of Agrin-dependent AChR aggregates therefore remains unexplained. Similar observations were reported in a previous study in which myotubes were treated with a human anti-MuSK mAb derived from patients with MuSK MG (30). We can hypothesize here that the anti-MuSK^CRD^ antibodies binding to MuSK are responsible for MuSK cross-linking, allowing the transphosphorylation of this protein to a level below the threshold required for efficient AChR clustering. However, as the intracellular domain of MuSK contains 19 tyrosine residues and phosphorylation of only the membrane-apposed Y553 and activation loop Y750, 754, and 755 residues is required for full MuSK activation (31–33), we cannot exclude the possibility that anti-MuSK^CRD^ antibodies trigger a change in MuSK conformation, exposing tyrosine residues that are not involved in MuSK signaling and allowing their phosphorylation, raising questions about the relevance of the 4G10 antibody for assessing MuSK activity accurately.

In addition to the core Agrin/LRP4MuSK signaling pathway, other accessory components, such as Wnt morphogens, have been shown to trigger MuSK activation via binding to its CRD, albeit to a lesser extent (12). We cannot rule out the possibility that anti-MuSK^CRD^ antibodies disrupt Wnt/MuSK interactions, but it remains unclear whether a loss of Wnt signals in adulthood makes a significant contribution to the observed phenotype. Many studies have demonstrated the crucial role played by Wnt pathways in the early steps of neuromuscular synaptogenesis at the NMJ (10, 11, 14). However, beyond the role of these pathways in prepatterning and synaptic contacts, their impact on NMJ maturation and maintenance remains poorly understood, with Agrin/MuSK signaling still largely considered to be the prevailing molecular mechanism responsible for ensuring synapse integrity (33). In the context of the passive transfer of anti-MuSK^CRD^ antibodies in adult animals, any change in Wnt signals is likely to be masked by the disruption of the core Agrin/MuSK signaling pathway, resulting in the severe NMJ phenotype observed.

It is important to take into account the discrepancies between human disease and animal EAMG models when considering the mechanisms involved in generating the phenotype. MuSK MG in humans is characterized by a prevalent impact of IgG4 immunoglobulins, which are unable to interact with complement components (34, 35). However, increasing evidence suggests that IgG1–3, capable of mediating a complement-dependent response, can play a pathogenic role in MuSK-MG onset (36, 37). Our passive transfer model was based on injections of IgG from rabbits immunized with the MuSK CRD peptide. Humans and mice have a broad diversity of IgG subclasses, whereas rabbits have only one IgG subclass, which interacts with Fc receptors and the complement molecule C1q (38). The expected effect of anti-MuSK^CRD^ antibodies, blocking the MuSK-Lrp4 interaction, was observed following the exposition to rabbit Ig, just as in the human disease, but we cannot rule out the involvement of synaptic degenerative activity due to complement activation, which may contribute to the disrupted NMJ phenotype in our passive transfer model. This hypothesis is also supported by isotyping experiments in our active transfer mouse model resulting in the development of complement-binding IgG2b antibodies after immunization with the MuSK^CRD^ peptide. This observation is consistent with the identification of anti-MuSK antibodies of the IgG2b subclass in previous mouse models of MG based on active immunization with the MuSK extracellular domain from rats or humans (27, 39, 40). Another intrinsic property of human anti-MuSK IgG4 antibodies is their ability to exchange Fab arms with other IgG4 antibodies, resulting in functionally monovalent antibodies (41, 42). This specific feature of IgG4 in humans plays a critical role in the pathogenic impact of antibodies targeting MuSK. However, to our knowledge, no study has ever reported the occurrence of such a process for rabbit IgG or mouse IgG1 or 2b. The polyclonal nature and intrinsic properties of the rabbit IgG against MuSK^CRD^ used in this study may underlie multiple effects. Our results provide strong evidence in support of the pathogenicity of antibodies targeting MuSK CRD, but further studies are required to decipher the precise molecular effects of this immunoreactivity in humans.

The involvement of the MuSK CRD in NMJ formation and physiology has recently been investigated in numerous studies, but immunoreactivity to this particular domain in the context of MG has rarely been considered (20–22). Therapeutic approaches for restoring NMJ integrity through the injection of agonist mAbs targeting the MuSK CRD have been developed in animal models of neuromuscular disorders (43–45). By contrast, the immunization of mice with a functionally monovalent mAb directed against the MuSK Ig1 domain results in a severe MG phenotype (46). Meta-analysis of the existing passive-transfer models of MuSK MG suggested that the pathogenicity of the injected Ig depends strongly on the epitope targeted, antibody titer, isotype, clonality, and functional valency (25). It is likely that a fraction of the CRD-targeting antibodies we generated induce an agonist effect similar to what is observed in anti-MuSK mAb-based therapies. However, the MG phenotype developed during our passive-transfer protocol indicates that the cumulative effects of the several epitopes targeted by the injected anti-MuSK^CRD^ tips the balance toward a pathogenic role. In summary, the unique NMJ features induced by the anti-MuSK^CRD^ antibodies used in this study reproduce the targeting of several epitopes occurring in patients with MuSK MG more effectively as opposed to the injection of a mAb. This study highlights the pathogenic potential of antibodies against the MuSK CRD in MG and provides a deeper understanding of the underlying pathophysiological molecular mechanisms, which are critical for the development of effective personal treatment.

## Methods

### Sex as a biological variable.

Our work focused solely on female mice because MG is reported to be more frequent in female patients. It is likely that the findings are relevant for male mice.

### MuSK^CRD^ peptide purification.

A cDNA construct encoding a fusion protein composed of a signal peptide, a 6x histidine tag, a thioredoxin fusion tag, and a tobacco etch virus (TEV) protease cleavage site followed by the human MuSK (NCBI, accession number NM_005592.4) frizzled-like CRD (amino acids 305–482) was produced by GenScript. The cassette was subsequently subcloned into a pCS2+ vector as a *Bam*HI/*Xho*I insert, and the plasmid was verified by sequencing. This construct was used to transfect HEK293T (ATCC) cells. The fusion protein produced in these cells was purified in 2 steps. After column equilibration, the supernatant was applied to a Ni-affinity HisTrap Excel column (17-3712-05, GE Healthcare), and the column was washed with 50 mM Tris-HCl pH 7.5/250 mM NaCl/10 mM imidazole. The protein was eluted in 50 mM Tris-HCl pH 7.5/250 mM NaCl/500 mM imidazole. The fractions containing the fusion product were pooled, concentrated on centrifugal concentrators with a 5 kDa cutoff (Sartorius), and further purified by size exclusion chromatography on Superdex 75 HR 10/300 columns (17517401, GE Healthcare). The purified fusion protein was cleaved by a recombinant TEV protease: His6-TEV(S219V)-Arg (ATCC) in 50 mM Tris-HCl pH 7.5/250 mM NaCl at 10°C overnight. A second round of purification (Ni affinity chromatography and gel filtration, as above) was performed to obtain a free MuSK^CRD^ peptide with a molecular weight of 16.4 kDa. Fractions of interest were pooled, and the purified protein was analyzed by SDS-PAGE. MuSK^CRD^ identity was confirmed by mass spectrometry (data not shown). A final concentration step was performed, and aliquots with a concentration of approximately 1 mg/mL were collected.

### Production of rabbit anti-MuSK^CRD^ antibodies.

Rabbits were immunized by the injection of 200 μg mouse recombinant MuSK^CRD^ peptide, followed by booster injections on days 7, 21, and 56 with another 200 μg of the peptide at each time point. Total IgGs were purified in accordance with Agro-Bio guidelines. Two of the 4 rabbits receiving these injections developed clinical signs of muscle weakness (data not shown). For the generation of control rabbit IgG, rabbits were injected using the same protocol, but preparations were free of MuSK^CRD^ peptide.

### Mouse experimental models.

Mice were kept at an animal facility under a 12-hour light/12-hour dark cycle, with access to standard rodent food and water ad libitum. Animals were allowed to acclimate to conditions in the facility for 1 week and to the various clinical tests to be performed before induction of the experimental models.

For passive antibody transfer, 23 8-week-old female B6/D2F1 mice (Charles River Laboratories) received daily i.p. injections for 20 days with 200 μL PBS (PBS group, *n* = 8) or 10 mg total IgGs diluted in 200 μL PBS, either from rabbits immunized with the MuSK^CRD^ peptide (CRD group, *n* = 8) or with a control preparation (IgG, *n* = 7). The first injection (day 0) was supplemented with 300 mg/kg cyclophosphamide monohydrate (218707, Sigma-Aldrich) to inhibit immune responses directed against the injected rabbit IgGs. Body weight was assessed every 4 days, and animals underwent clinical tests and blood collection on days –2, 4, 12, and 19.

For active immunization with the MuSK^CRD^ peptide, 16 8-week-old female C57BL/6 mice (Charles River Laboratories) were anesthetized with a ketamine/xylazine cocktail. They then received an injection of 30 μg mouse recombinant MuSK^CRD^ protein emulsified in a 200 μL solution composed of 50% CFA (F5881, Sigma-Aldrich) supplemented with 2 mg inactivated *Mycobacterium tuberculosis* and made up to the required volume with saline. Equal quantities of the solution were injected into each hindlimb footpad and subcutaneously at 3 sites on the back. Booster injections of 30 μg MuSK^CRD^ peptide emulsified in a 200 μL solution of 50% incomplete Freund adjuvant (F5506, Sigma-Aldrich) and 50% saline were administered on days 21, 42, and 59 via subcutaneous injections of equal volumes at 3 sites on the back (acCRD group, *n* = 16). In parallel, 10 mice received similar injections without the recombinant MuSK^CRD^ protein as a control (CFA group, *n* = 10). Body weight was assessed weekly, and animals were subjected to clinical tests and blood collection every 2 weeks.

### Clinical assessment of myasthenic symptoms.

We subjected the mice to a repeated battery of clinical tests during the experimental procedure to study the onset of muscle weakness and to grade the animals in terms of symptom intensity, as described by Weiss et al. (47). Body weight was measured, compared with the mean value for the control group (PBS or CFA group), and the animal was given a score out of 3. The animals were then gently dragged 10 times over a grid and placed in the middle of the grid, which was then immediately inverted. The behavior of the mice while hanging upside down was observed over a period of 60 seconds. This behavior and the time spent hanging upside down from the inverted grid accounted for another 3 points. Finally, mice were subjected to strength analysis. They were allowed to run for 5 minutes on a treadmill, then held by the tail and allowed to grab a bar connected to a force transducer. The animals were then gently pulled horizontally until the loss of grip. Comparisons of strength between control and immunized animals were used as the basis for the attribution of a final grade on a 3-point scale. By adding the 3 scores for these clinical tests together, we obtained a global clinical score on a scale of 0 to 9. Higher scores on this scale were associated with worse symptoms.

### Electromyography and in situ muscle strength measurements.

In situ muscle strength and CMAP recordings were performed as previously described (48). Briefly, for isometric force analysis, tetanic stimulation of the sciatic nerve and muscle was performed for 500 ms at 100 Hz and optimal tibialis anterior muscle stretching parameters, that is, when the recorded force is maximal for an isometric contraction. For CMAP recordings, the sciatic nerve was stimulated with a multielectrode setup, as suggested in a previous study (49). CMAPs were recorded for stimulations at frequencies ranging from 1 to 40 Hz, with trains of 10 stimulations separated by 1-minute rest periods.

### ELISA.

ELISAs were performed on the serum samples obtained after each clinical test and when the animals were euthanized. Nunc MaxiSorp flat-bottomed 96-well plates (44-2404-21, Thermo Fisher Scientific) were coated by incubation overnight at 4°C with 1 μg/mL recombinant MuSK^CRD^ protein diluted in 10 mM NaHCO_3_ coating buffer. The plates were then washed 3 times with PBS/0.05% Tween 20 and blocked by incubation with PBS/2% BSA at 37°C for 2.5 hours. After 3 washes in PBS/0.5% Tween 20, the plates were incubated with serum samples with dilution factors in PBS/0.1% BSA ranging from 1:500 to 1:1,000 (active immunization model) to 1:100,000 (passive transfer model) for 90 minutes at 37°C. The plates were washed several times and incubated for 60 minutes at 37°C with HRP-conjugated or biotin-conjugated secondary antibodies: goat anti-rabbit IgG HRP (31466, Invitrogen), goat anti-mouse IgG HRP (1:10,000, 31431, Invitrogen), biotin-rat anti-mouse Ig1 (1:250, 553441, BD Biosciences), and biotin-rat anti-mouse Ig2b (1:250, 553393, BD Biosciences) in PBS/0.1% BSA. If necessary, the plates were then incubated with Streptavidin-HRP conjugate (S911, Thermo Fisher Scientific) diluted 1:10,000 in PBS/0.1% BSA for 30 minutes. The plates were subjected to a final series of washes and then incubated with tetramethylbenzidine (421101, BioLegend) for colorimetric reaction. The reaction was stopped by adding 1 M phosphoric acid, and optical density was measured at 450 nm with a Spark 10M microplate reader (TECAN Life Sciences).

### Immunostaining of isolated muscle fibers and cryosections.

Tibialis anterior muscles were dissected, rinsed once in 1× PBS, and fixed by incubation in PBS/4% PFA at room temperature for 1 hour. Groups of 10–20 muscle fibers were gently dissected from the whole muscle and incubated overnight in 0.1 M glycine in PBS at 4°C with mild shaking. The fibers were washed several times in PBS, permeabilized, and blocked by incubation for 4 hours in PBS/4% BSA/5% goat serum/0.5% Triton X-100 at room temperature. The fibers were then incubated with the following primary antibodies in blocking solution for 48 hours at 4°C: rabbit polyclonal anti-synaptic vesicle protein 2A antibody (1:100, SV2, DSHB) or rabbit polyclonal anti-synaptophysin antibody (1:100, 180130, Thermo Fisher Scientific) and rabbit polyclonal antineurofilament light chain (1:500, AB9568, Merck Millipore). The fibers were then rinsed for several hours at room temperature in 0.5% Triton X-100 in PBS and incubated overnight at 4°C with Alexa Fluor 594–conjugated goat anti-rabbit antibody (1:400, A32740, Thermo Fisher Scientific) and Alexa Fluor 488–conjugated α-bungarotoxin (1:500, B13423, Thermo Fisher Scientific) in blocking solution. Finally, the fibers were washed for 1 day every hour with 0.5% Triton X-100 in PBS and mounted on slides in nonhardening Vectashield mounting medium (H-1000, Vector Laboratories).

### Cell culture, transfection, and treatments.

Primary myoblasts from WT and transgenic MuSKΔCRD mouse models (14), HEK293T cells (ATCC), COS7 cells (ATCC), and C2C12 myoblasts (ATCC) were maintained and differentiated, if necessary, as previously described (48). For transfection, COS7 cells were used to seed 10 cm round dishes and were grown to 70%–80% confluence. Next, 2 μg of MuSK-FLAG (Lin Mei, Case Western Reserve University, Cleveland, Ohio, USA) (6) and/or Lrp4-HA coding plasmid (50) were diluted in OptiMEM medium (Gibco, Life Technologies) for 15 minutes at room temperature in the presence of FuGENE6 transfection reagent (E2691, Promega). Next, 80% of the cell medium was replaced with DMEM (Gibco) alone or DMEM supplemented with 1% normal rabbit serum or MuSK^CRD^-immunized rabbit serum. The DNA mixture was then added to the cells, which were incubated for 24 hours to allow the production of sufficient recombinant protein. For MuSK^CRD^ peptide production, adherent HEK293T cells were transfected 24 hours after plating in 24 μL polyethylenimine (1 mg/mL, Polysciences Inc.) with 12 μg pCS2+ MUSK CRD mixed with 400 μL of serum-free DMEM. The culture medium was replaced, and after 15 minutes of incubation at room temperature, the mixture was added dropwise to each plate. After 24 hours, the cell culture medium was replaced with OptiMEM medium supplemented with 0.5% FBS. Culture supernatants were collected 48–96 hours after transfection, filtered, and stored at −80°C until purification.

### Staining of the AChR cluster in vitro.

Primary myoblasts from WT and MuSKΔCRD (14) mice were allowed to differentiate into myotubes and were then treated with 0.4 μg/mL recombinant rat Agrin (550-AG-100/CF, R&D Systems) and 1% control rabbit serum (16120-099, Gibco, Life Technologies) or MuSK^CRD^-immunized rabbit serum for 16 hours. Cells were rinsed twice with 1× PBS and incubated with PBS/4% PFA for 20 minutes. They were washed twice in PBS and then incubated with tetramethylrhodamine-conjugated α-bungarotoxin (1:1,000, T1175, Thermo Fisher Scientific) for 1 hour at room temperature. The cells were rinsed twice in PBS and incubated for 5 minutes with Hoechst stain (33342, Thermo Fisher Scientific) diluted to 0.5 μg/mL in PBS to label nuclei. After a final wash in PBS, the cells were mounted in Vectashield H-1000 mounting medium.

### Cell surface biotinylation.

C2C12 myotubes were rinsed 3 times with ice-cold 0.1 mM CaCl_2_/1 mM MgCl_2_ in PBS (PBS Ca Mg) and were then incubated with 0.5 mg/mL EZ-Link Sulfo-NHS-SS-Biotin (21331, Thermo Fisher Scientific) in PBS Ca Mg for 20 minutes at 4°C to label surface proteins. Free biotin was quenched by 3 washes in PBS Ca Mg supplemented with 50 mM glycine, and the cells were then rinsed twice with PBS Ca Mg and once with calcium- and magnesium-free PBS. Cells were lysed by incubation in a specific lysis buffer consisting of 50 mM Tris HCl/150 mM NaCl/2 mM EDTA/1% Triton X-100 plus protease inhibitors for 20 minutes at 4°C. Cells were scraped off the dish and the lysates were centrifuged at 20,000*g* and 4°C for 20 minutes. The supernatant was collected, and a small fraction was retained for input analysis. The remaining volume was incubated with Pierce High-Capacity Streptavidin Agarose (20357, Thermo Fisher Scientific) overnight with end-to-end rotation in a cold room. The complexed proteins were then washed 3 times with Pierce RIPA buffer (89901, Thermo Fisher Scientific) supplemented with protease inhibitors (78438, Thermo Fisher Scientific) and once with 50 mM Tris supplemented with protease inhibitors. They were then eluted in 4× Laemmli buffer at 95°C for 5 minutes.

### IP.

Cell protein extracts from COS7, C2C12, WT, and MuSKΔCRD primary myotubes were obtained as previously described (51). Lysates were precleared by incubation with SureBeads Protein G Magnetic Beads (1614023, Bio-Rad) for 1 hour at 4°C with end-to-end rotation. The supernatants were then incubated overnight at 4°C with the following antibodies for IP: rat IgG isotype control (31933, Invitrogen), rabbit polyclonal anti-MuSK (ABS549, Merck Millipore), or serum from MuSK^CRD^-immunized rabbits. The samples were then incubated with magnetic beads supplemented with protease inhibitors and phosphatase inhibitors (78426, Thermo Fisher Scientific) for 4 hours at 4°C with end-to-end rotation. The beads were collected and washed 3 times in RIPA and once in 50 mM Tris-HCl pH 8, both supplemented with protease inhibitors and phosphatase inhibitors. Finally, IP proteins were eluted in 4× Laemmli buffer at 95°C for 5 minutes. For IP of FLAG-tagged transfected proteins, we used the DYKDDDDK Fab-Trap Agarose Kit (ffak-20, Chromotek, Proteintech) in accordance with the manufacturer’s instructions. Complexes were collected by competitive elution with the 3xDYKDDDDK peptide (fp-1, Chromotek, Proteintech).

### Western blotting.

Cell proteins were extracted and SDS-PAGE was performed as previously described (51). Samples were transferred overnight onto an Immuno-Blot PVDF membrane (1620177, Bio-Rad). The membrane was blocked in TBS/0.1% Tween 20 (TBST) supplemented with 5% BSA or 5% nonfat milk powder, depending on the specifications of the primary antibody. The membrane was then incubated overnight at 4°C with mild shaking with the following primary antibodies diluted in the appropriate buffer: rabbit polyclonal anti-HA (1:5,000, ab9110, Abcam), rabbit polyclonal anti-MuSK (1:500, ABS549, Merck Millipore), mouse monoclonal anti-GAPDH (1:4,000, clone 6C5, sc-32233, Santa Cruz Biotechnology), mouse monoclonal anti-transferrin receptor (1:1,000, clone H68.4, 13-6800, Invitrogen), mouse monoclonal anti-FLAG (1:1,000–1:10,000, clone M2, F1804, Sigma-Aldrich), and mouse monoclonal cocktail 4G10 platinum anti-phosphotyrosine (1:1,000, 05-1050, Sigma-Aldrich). The membrane was washed several times in TBST and then incubated for 1 hour at room temperature with the following secondary antibodies: StarBright Blue 700 goat anti-mouse and anti-rabbit antibodies (1:10,000, 12004158 and 12004161, respectively, Bio-Rad). Signals were observed and digitized with the ChemiDoc MP imaging system (Bio-Rad), and signal intensity was quantified with Fiji software and Gels tool.

### Image acquisition.

All images were acquired with a Zeiss Axio Imager M2 microscope equipped with an ApoTome 2 module, using a Plan-Apochromat 40×/1.3 NA oil DIC objective or a Zeiss LSM-880 confocal laser scanning microscope using a Plan-Apochromat 63×/1.4 NA oil DIC objective. Image acquisition was performed with ZEN 3.5 Blue edition software (Zeiss) on sets of images taken in multiple focal planes separated by a regular interval (*Z*-stacks). Images were then analyzed and presented as maximum intensity projections derived from *Z*-stacks. Similar acquisition parameters were used for each series of images from the same experiment to ensure relevant comparisons.

### Statistics.

We first tested the normality of data distribution with the Shapiro-Wilk test and then performed appropriate 2-tailed parametric tests or their nonparametric equivalents, including Student’s *t* test, Mann-Whitney *U* test, 1-way ANOVA with Tukey’s post hoc test, and 2-way ANOVA with Tukey’s post hoc test. All results are expressed as mean ± SEM. Differences with *P* values below the α-risk threshold of 0.05 were considered significant, and trends (*P* < 0.1) are indicated when relevant. No outlier was removed. In figure panels with indication of significance in a stacked fashion, black symbols refer to the CRD versus PBS comparison, whereas gray symbols refer to the CRD versus IgG comparison.

### Study approval.

All experiments were performed in accordance with European and national guidelines for animal experimentation with the approval of the institutional ethics committee (A1-0018).

### Data availability.

All individual values represented in graphs or used to calculate means are compiled in the Supporting Data Values file published with this article.

## Author contributions

MH, LS, RLP, BF, NS, MM, and JM conceived the study and designed the methodology. LS and MH wrote the manuscript. MH performed most of the experiments and analyzed all the data. SC, CB, AY, AG, and LSL contributed to animal experimentation and biochemistry. ML performed the electrophysiological recordings. JE, YR, NS, and MM conceived and performed MuSK^CRD^ peptide purification. SC, CB, AG, and CI contributed to cell culture. All authors read, proofread, and accepted the manuscript.

## Supplementary Material

Unedited blot and gel images

Supporting data values

## Version 1 06/12/2025

In-Press Preview

## Version 2 08/01/2025

Electronic publication
