# Supplementary material for: MuSK cysteine-rich domain antibodies are pathogenic in a mouse model of autoimmune myasthenia gravis
Source: J Clin Invest. 2025 Jun 12;135(15):e173308. doi: 10.1172/JCI173308 (PMC12321381; doi:10.1172/JCI173308)

Full unedited blot for Figure 1

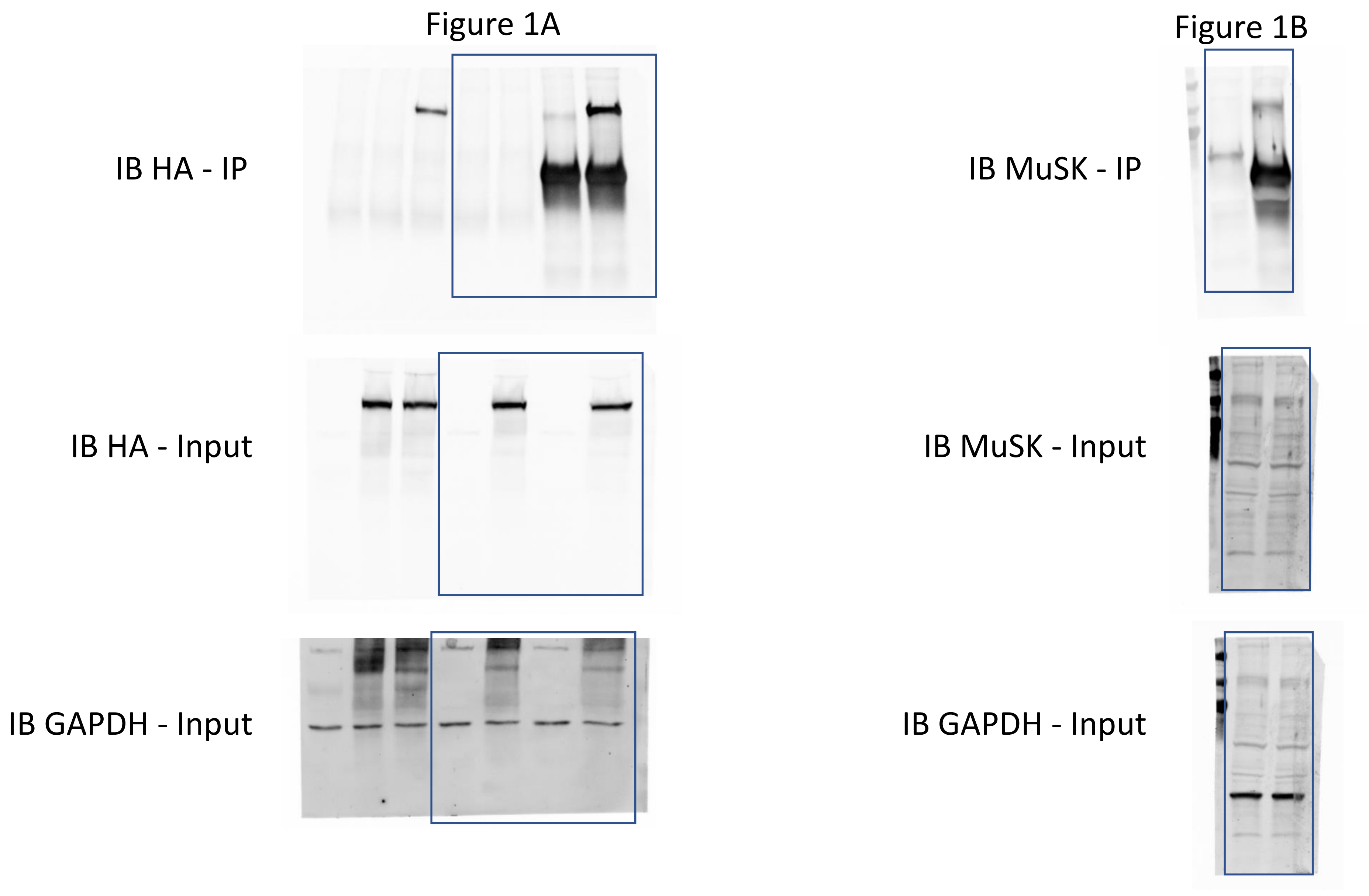

Full unedited blot for Figure 4

Figure 4C

IB MuSK - IP

IB MuSK - Input

IB GAPDH - Input

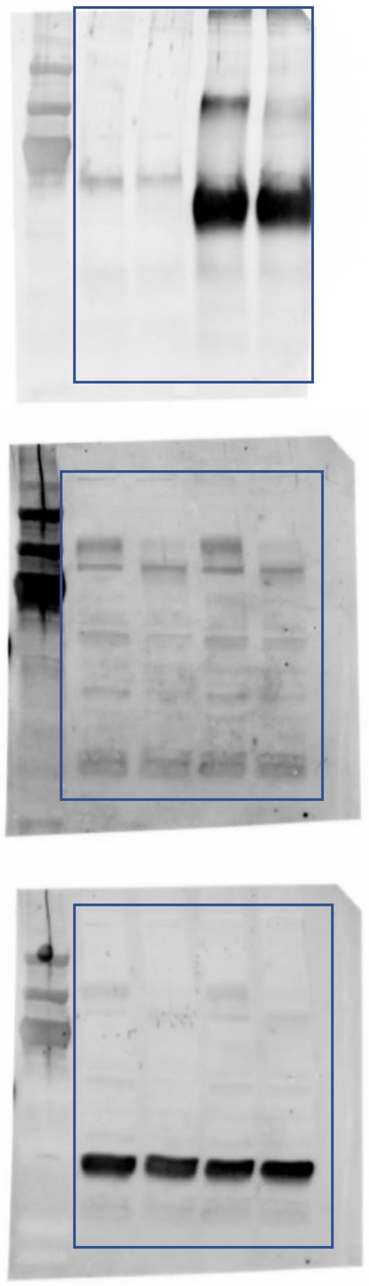

Full unedited blot for Figure 5

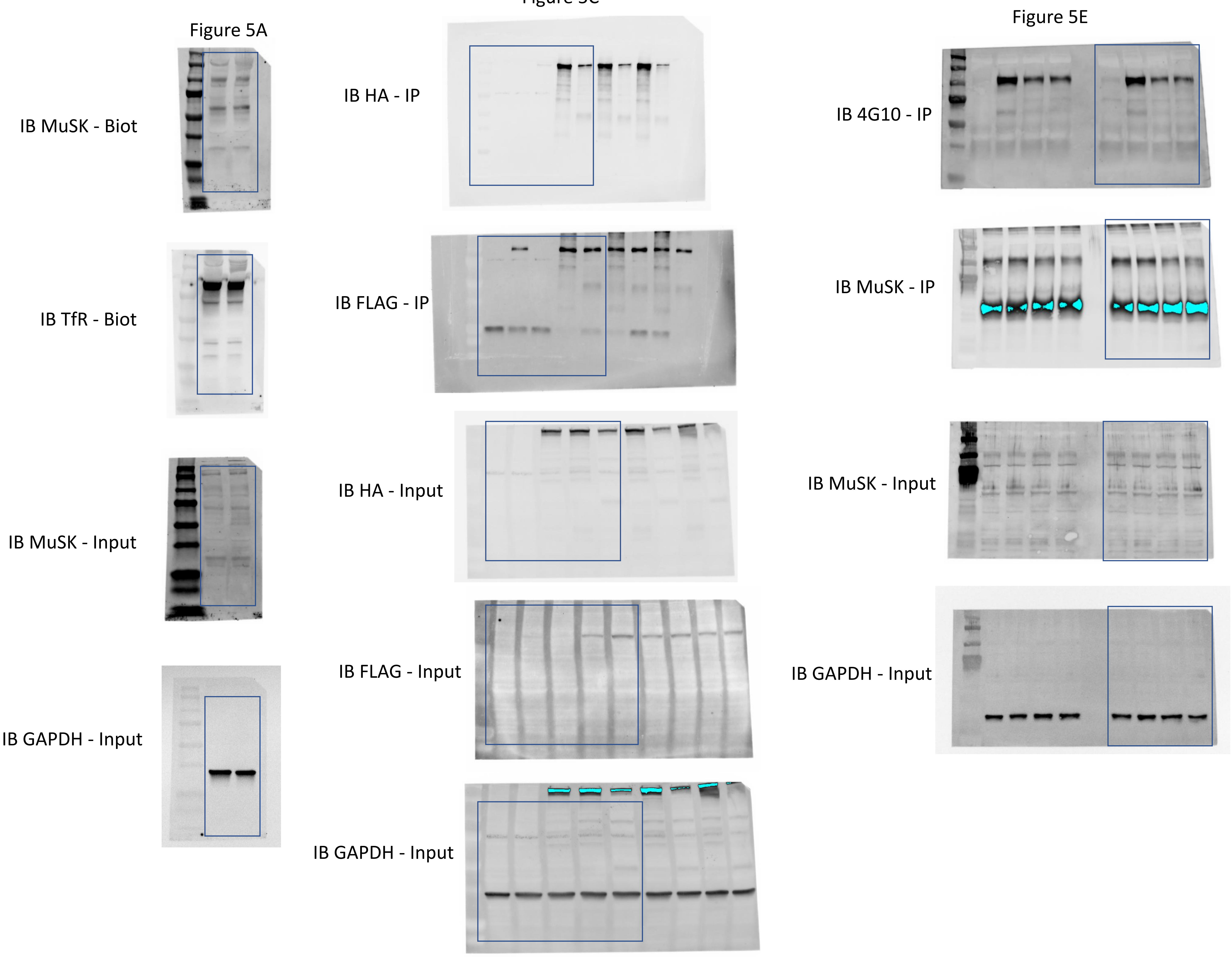

Supplement: Unedited blot and gel images [file jci-135-173308-s077.pdf]
